# The Life Histories of Intermediate Hosts and Parasites of *Schistosoma haematobium* and *Schistosoma mansoni* in the White Nile River, Sudan

**DOI:** 10.3390/ijerph19031508

**Published:** 2022-01-28

**Authors:** Hassan Ahmed Hassan Ahmed Ismail, Abed el Aziz Abed el Rahim Mohamed Ahmed, Seungman Cha, Yan Jin

**Affiliations:** 1 Communicable and Non-Communicable Diseases Control Directorate, Federal Ministry of Health, Khartoum 1111, Sudan; hassanhassoon@hotmail.com; 2 Schistosomiasis Research Laboratory, Zoology Department, Faculty of Science, University of Khartoum, Khartoum 11111, Sudan; abdellazizz@gmail.com; 3 Department of Global Development and Entrepreneurship, Graduate School of Global Development and Entrepreneurship, Handong Global University, Pohang 37554, Korea; seungman.cha@handong.edu; 4 Department of Disease Control, London School of Hygiene & Tropical Medicine, London WC1E 7HT, UK; 5 Department of Microbiology, College of Medicine, Dongguk University, Gyeongju 38066, Korea

**Keywords:** *Biomphalaria pfeifferi*, *Bulinus truncatus*, cercarial rhythmicity, survival rate, cumulative hazard ratio, snail, Sudan, schistosomiasis

## Abstract

Background: The epidemiology of schistosomiasis transmission varies depending on the circumstances of the surrounding water bodies and human behaviors. We aimed to explore cercarial emergence patterns from snails that are naturally affected by human schistosomiasis and non-human trematodes. In addition, this study aimed to explore how schistosomiasis infection affects snail survival, reproduction, and growth. Methods: We measured the survival rate, fecundity, and size of *Biomphalaria pfeifferi* snails and the cercarial rhythmicity of *S. haematobium* and *S. mansoni*. The number of egg masses, eggs per egg mass, and snail deaths were counted for 7 weeks. The survival rate and cumulative hazard were assessed for infected and non-infected snails. Results: *S. haematobium* and *S. mansoni* cercariae peaked at 9:00–11:00 a.m. Infection significantly reduced the survival rate of *B. pfeifferi*, which was 35% and 51% for infected and non-infected snails, respectively (*p* = 0.02), at 7 weeks after infection. The hazard ratio of death for infected snails compared to non-infected snails was 1.65 (95% confidence interval: 1.35–1.99; *p* = 0.01). Conclusions: An understanding of the dynamics of schistosomiasis transmission will be helpful for formulating schistosomiasis control and elimination strategies. Cercarial rhythmicity can be reflected in health education, and the reproduction and survival rate of infected snails can be used as parameters for developing disease modeling.

## 1. Introduction

Schistosomiasis, an acute and chronic parasitic disease, is a major public health issue in 51 endemic countries, with moderate-to-high transmission [1]. Despite global control efforts for schistosomiasis, it is estimated that fewer than 50% of the 237 million people who required preventive treatment in 2019 received treatment [1]. The overwhelming majority (211 million people) of those who need preventive chemotherapy live in Africa. Estimates of schistosomiasis-specific deaths per year across the world range from 24,072 to 200,000 [2,3,4]. Schistosomiasis-specific deaths are substantially concentrated in low- and low-middle-income countries (94%) [2]. As is the case for many other neglected tropical diseases, schistosomiasis is a disease of poverty since infection transmission takes place where poverty, malnutrition, inadequate sanitation, and lack of safe water prevail [3]. Rural inhabitants in endemic countries, who are most at risk of schistosomiasis, are in many cases co-infected with other parasites, such as soil-transmitted helminthiasis, largely because of their poor living conditions [5,6,7]. School-age children experience especially detrimental effects from schistosomiasis, including anemia, malnutrition, stunted growth, and impaired cognitive development [7,8,9,10,11,12]. Schistosomiasis also has a negative impact on workers’ quality of life and productivity [13,14]. However, for many individuals, contact with infested water is unavoidable as part of their routine agricultural, domestic, occupational, and recreational activities [15,16].

The transmission of schistosomiasis in humans is a complex process consisting of a vicious cycle of events, wherein humans are definitive hosts of adult schistosomes [17]. In this regard, it is critical to clearly understand the epidemiology of parasites and their intermediate hosts. *Schistosoma* parasites are found as adult worms in the vascular system of humans and other mammals. The eggs produced by adult worms of *S. haematobium* or *S. mansoni* are excreted in urine or stool. The eggs hatch in water under optimal conditions and release swimming miracidia, which locate and penetrate freshwater snails (*Biomphalaria* and *Bulinus*), the intermediate hosts of schistosomiasis.

Although many studies have suggested diurnal or nocturnal patterns of cercaria emergence, limited research has comprehensively investigated the cercarial rhythmicity of schistosomes, comparing mortality, fecundity, and growth between infected and non-infected snails in Sudan [18,19,20,21]. In Sudan, schistosomiasis is often associated with water resource development projects, particularly irrigation schemes, where snails (the intermediate hosts of the parasite) breed [22]. The characteristics of cercarial emergence patterns, the life-history traits of *Schistosoma*, and their interactions with snails have not been well documented in Sudan. Thus, it is important to understand cercarial release patterns and the influence of schistosomiasis infection on snails, and this information can be incorporated into schistosomiasis-elimination strategies in Sudan.

We aimed to explore cercarial emergence patterns from snails that are naturally affected by human schistosomiasis (*Bulinus truncatus* and *Biomphalaria pfeifferi*) and non-human trematodes, collected from the White Nile River in Sudan. In addition, this study aimed to explore how schistosomiasis infection affects snail survival, reproduction, and growth by comparing the survival rate, fecundity, and size between infected and non-infected snails. This is a follow-up study after our earlier longitudinal malacological research, which was conducted for 1 year at three different shore sites in the vicinity of El Shajara along the White Nile River in Khartoum State, Sudan [23]. Most of the collected snails were *B. pfeifferi, B. truncates, Physa acuta,* and *Melanoides tuberculata*. The first study involved field-based observational research, whereas this follow-up, laboratory-based study included an experiment comparing infected and non-infected snails.

## 2. Materials and Methods

### 2.1. Collection and Identification of Snails

The collected snails were pooled in plastic containers filled with water. Within 2–3 h after collection, all snails were cleaned. First, we placed the collected snails into a plastic container, gently rinsed them with tab water, and poured out all the mud in the plastic container through a plastic mesh scoop strainer sieve. We repeated this procedure three to four times. Finally, we removed grasses, plant leaves, algae, and debris with fine-tipped forceps. They were then identified and classified by species based on the criteria of the Field Guide to African Freshwater Snails of the Danish Bilharziasis Laboratory [24]. We used a dissecting microscope to identify snail type. We found 4243 *Cleopatra bulimoides*, 1076 *Melanoides tuberculata*, 2425 *Physa acuta*, and 757 *Pila ovata snails* in addition to 5408 *B. pfeifferi* and 3893 *B. truncatus snails*. No *B. sudanica* snails were observed. Of the *B. pfeifferi* and *B. truncatus* snails, 44 and 53 were infected with *S. haematobium* and *S. mansoni*, respectively. We observed the infected snails in March–June, which is classified as the dry season and early rainy season in Sudan. Out of 5408 *B. pfeifferi* snails, 31 naturally liberated other trematodes’ cercariae (infection rate: 0.6%), and 57 *B. truncatus* snails out of 3893 shed other trematodes’ cercariae (1.5%). More specifically, out of 3893 *B. truncates* snails, 21 released echinostome and 36 shed amphistome cercariae. Out of 5408 *B. pfeifferi* snails, 20 released echinostome cercariae and 11 shed amphistome cercariae.

### 2.2. Natural Infections of the Snails

Natural infections in the snails were examined from the day of collection for a 1-month period using the natural emergence technique [25]. Clean snails were separated in groups. Each group of 20 snails was transferred to a small beaker containing 200 mL of dechlorinated tap water. They were placed under artificial illumination at a temperature of 20–25 °C for an interval of 4 to 6 h starting at 8:00 a.m. The emergence of cercariae was checked at regular short intervals since emerged cercariae might encyst onto external substrates or sometimes re-enter the snails quickly. If any beaker was confirmed to contain emerged cercariae, the snails in that beaker were subsequently transferred to small glass bottles for individual screening. This procedure was repeated by placing each snail into the bottle one by one to check their infection status. The number of snails shedding cercariae was recorded, and those shedding no cercariae were reexamined on a weekly basis using the same procedure for a 1-month period. The details of the methods used to assess the infection status of snails, and the results have been previously published. We used some of the naturally infected snails to explore the rhythmicity.

### 2.3. Identification of Cercariae

Identification of the emerged cercariae at the major type level was carried out using living (unstained) cercarial specimens that were transferred to glass slides. In this technique, cercariae were taken from the beaker where they emerged using a pipette and transferred in a drop of water onto a glass slide that was covered by a slip. Next, the excess water was carefully drawn off using a strip of filter paper that was applied to one side of the coverslip. The removal of excess water immobilized the cercariae and allowed some slight flattening of the cercariae, making their external and internal structures easier to observe.

Identification of cercariae at the sub-type, genus, and species level was carried out by applying staining methods using iodine and Ehrlich’s hematoxylin, with which their morphological features were easily observed. The trematode cercariae identification was conducted based on previously published morphological criteria [25]. The specific morphological features and the main diagnostic characteristics used to identify the cercarial specimens were their general appearance, tegument, body suckers, alimentary system, excretory system gland cells, genital primordium, and shape and relative dimensions of the cercarial tail. For identifying these morphological features and the main diagnostic characteristics, we took the following procedure: we removed a few cercariae from the emergence beaker using a pipette, transferred the cercariae in a drop of water onto a glass slide, carefully added a coverslip, and drew off excess water using a strip of filter paper applied to one side of the coverslip. The preparation was observed using a microscope (Olympus CX21).

### 2.4. Snail Maintenance and Breeding

The snails harboring the same type of trematode cercariae were separated into small plastic bowls with dechlorinated tap water and fed lettuce, and they were kept in darkness until their use for further examinations. After the 1-month examination, the uninfected snails were transferred for breeding indoors in plastic aquaria, which contained 10 L of dechlorinated tap water and were maintained at room temperature (25–35 °C). Snails were fed fresh lettuce every other day. The water in the aquaria was changed once a week.

### 2.5. Fecundity, Size, and Survival Rate of Snails

We measured the survival rate, fecundity, and size of *B. pfeifferi* snails. The number of egg masses and eggs per egg mass produced daily were counted for 7 weeks. The detailed methods are presented below.

#### 2.5.1. First Generation of Snails

When *B. pfeifferi* snails collected from the White Nile shore laid egg masses, the hatched neonates were transferred into new aquaria. We waited until they became mature snails of the same size, with a 6-mm diameter, for the study.

At the same time, stool samples were collected from all 76 students in the Quranic school (*khwala*) and were transferred to the Schistosomiasis Research Laboratory of the University of Khartoum. We examined the samples using a duplicate Kato–Katz (KK) smear and found 27 that were positive for *Schistosoma mansoni.* The 27 positive samples for *S. mansoni* were kept in the refrigerator (4 °C) to be used on the day of the experiment. The hard fecal samples were homogenized in pestle and mortar with the addition of some distilled water but not the mushy stool samples. The collected excreta were combined and macerated with a few drops of water until they became mushy. The material was then placed in several conical flasks. Cold water (10–15 °C) was added to the material in the flasks and allowed to precipitate. The obtained supernatant was changed many times using cold water until it was observed to be clear. Sediments that contained schistosome eggs were kept in the refrigerator for later usage as scheduled. The stored sediment was then taken using a Pasteur pipette into medium-sized Petri dishes (8 cm in diameter) that were half-filled with warm water (35–37 °C). The Petri dishes were exposed to artificial fluorescent light for 4 h (8:00 a.m. to 12:00 p.m.) to stimulate miracidial hatching. Using a binocular dissecting microscope, the successfully hatched miracidia were visualized actively swimming at the rim surface of Petri dishes.

#### 2.5.2. Infection of *B. pfeifferi* Snails

For experimental study assessing mortality, fecundity, and snail size, we chose 50 infected and 50 non-infected snails. Completely non-infected snails were used as the control group, and other snails infected only with *Schistosoma* were used as the experimental group. The non-infected snails were re-examined repeatedly to check their infection status on a weekly basis for a 4-week period to make sure they were completely non-infected. Each group of snails was maintained separately in plastic containers.

Warm water (32 °C) was added to the sediment. The funnel flask component was poured into glass Petri dishes. The Petri dishes were placed under strong artificial illumination. *S. mansoni* miracidia started to hatch and swam to the surface of the Petri dishes. A Pasteur pipette with a rubber bulb was used to pick up miracidia from the glass Petri dishes under dissecting microscopy, and 50 laboratory-bred *B. pfeifferi* snails were transferred to hemagglutination plates. One plate had 12 exposure units shaped like a well, and each unit contained 3–5 mL of freshwater. We placed one snail in each unit. Two miracidia were transferred to one exposure unit, and we covered the exposure unit with a sheet of glass. We waited until the snails were infected.

The snails were transferred to new plastic aquaria next day and maintained in the laboratory. They were fed fresh lettuce and examined every week for 7 weeks. We examined 50 other non-infected snails for the same period.

The numbers of egg masses and eggs produced per egg mass were counted weekly for 7 weeks. Snail size was measured by the shell width on a weekly basis during the 7-week period with calipers calibrated using Vernier scale measurements. To measure fecundity, we counted the number of the eggs and egg masses produced weekly by each snail. After every count on a weekly basis, we removed the egg masses and eggs from the plastic aquaria to ensure that they would not be included in the next count. To evaluate mortality, all dead snails were counted weekly for 7 weeks and then removed from the aquaria after counting.

### 2.6. Cercarial Rhythmicity

From each cercarial type, three sets of five naturally infected snails were placed in small beakers, to which 15 mL of warm water (32–37 °C) was added. The beakers were placed under natural light from 7:00 a.m. to 7:00 p.m. near a window. The water in each screening beaker was poured out every 2 h separately into a Petri dish. Each Petri dish was put under a dissecting microscope to count the number of harvested cercariae every 2 h. The cercarial suspension in the beaker was taken by a Finn pipette into small Petri dishes (50 μL each). A drop of iodine was added to kill and stain the cercariae, and then, they were counted under a dissecting microscope.

### 2.7. Ethical Clearance

Ethical approval was obtained on 9 March 2021 from the Department of Zoology Board and the Faculty of Science Scientific Research Board, Faculty of Science, the University of Khartum.

### 2.8. Statistical Analysis

We used Kaplan–Meier survival analysis and generated Kaplan–Meier survival curves and cumulative hazard graphs with 95% confidence intervals. To assess the significance of differences in the survival rate between infected and non-infected snails, we used the log-rank test. We ran Cox regression to explore the hazard ratios of infected snails compared to non-infected snails and used the *t*-test to assess differences in the fecundity of snails between the two groups. Due to the small sample size, a non-parametric analysis (the Mann–Whitney test) was used to investigate differences in snail size between the two groups. The statistical analysis was conducted using the R statistical software (R 4.1.0).

## 3. Results

### 3.1. Diurnal Rhythmicity and Production of Cercariae

**Figure 1:**
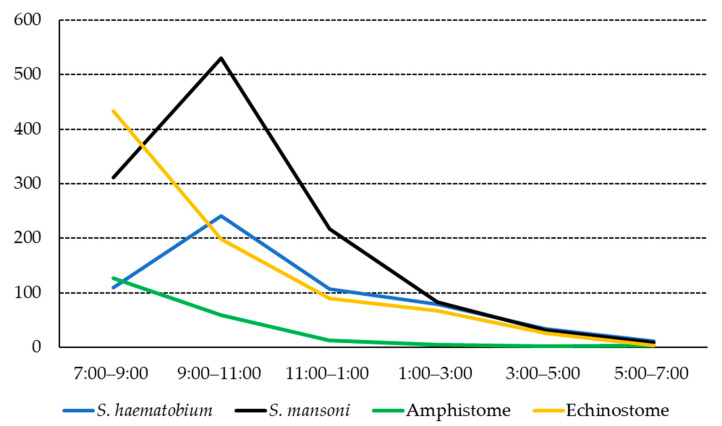
Overall diurnal innate rhythmicity of cercariae liberated from *Biomphalaria pfeifferi* and *Bulinus truncatus* snails (*y*-axis: number of cercariae).

Urinary human cercariae showed a remarkably consistent daily pattern. Their emergence began at sunrise, around 7:00 a.m. in Sudan Standard Time (SST). S. *haematobium* cercariae peaked at 9:00–11:00 a.m. and then steadily reduced until around sunset (7:00 p.m.). Amphistome cercariae commenced with a peak at 7:00–9:00 a.m. and then rapidly declined and completely disappeared around sunset. Very few amphistome cercariae were released in the afternoon from the naturally infected *B. truncatus* snails. Like urinary human cercariae, *S. mansoni* cercariae peaked at 9:00–11:00 a.m., followed by a rapid decline, and very few were released at 5:00–7:00 p.m. The echinostome cercariae emergence peaked at 7:00–9:00 a.m. and then started to decline and completely disappeared around sunset. Only a few amphistome cercariae were released after 5:00 p.m. from the infected *B. pfeifferi* snails. Figure 1 shows the cercarial release pattern from *B. truncatus* and *B. pfeifferi*.

### 3.2. Comparison of Survival, Fecundity, and Growth between Infected and Non-Infected Snails

**Figure 2:**
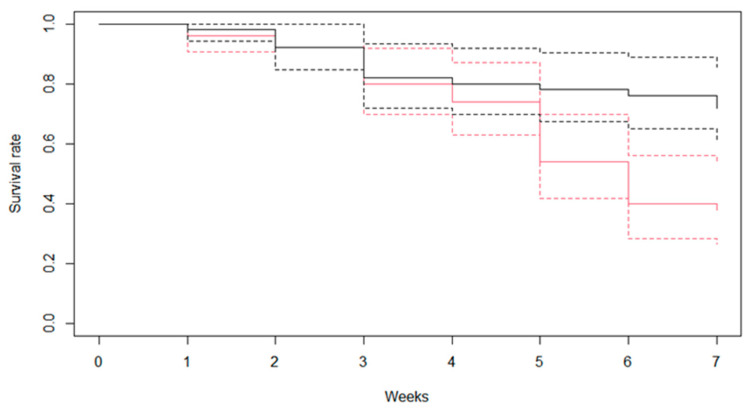
Weekly survival rates of *Biomphalaria pfeifferi* snails that were infected and non-infected with *Schistosoma mansoni* (black color, non-infected snails; red color, infected snails; dotted line, 95% confidence interval).

**Figure 3:**
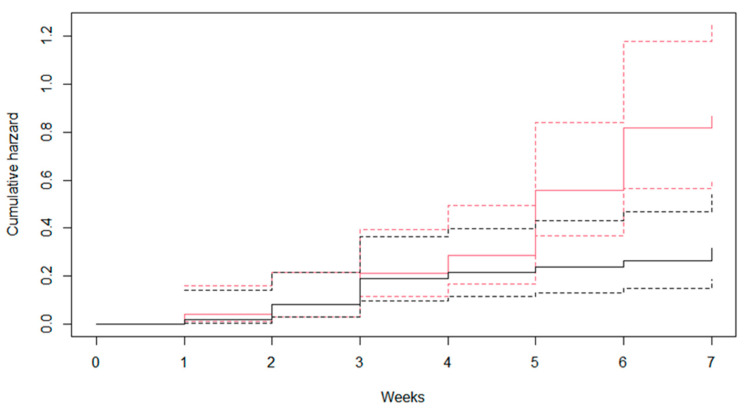
Cumulative hazard of *Biomphalaria pfeifferi* snails that were infected and non-infected with *Schistosoma mansoni* (black color, non-infected snails; red color, infected snails; dotted line, 95% confidence interval; cumulative hazard ratio, 1.65; 95% confidence interval, 1.35–1.99, *p* = 0.01).

Figure 2 shows the results of survival rates of *B. pfeifferi* snails infected and not infected with *S. mansoni*. The infection significantly reduced the survival rates of *B. pfeifferi*, which were 35% and 51% for infected and non-infected snails, respectively (*p* = 0.02) at 7 weeks after infection. The cumulative hazard is shown in Figure 3. The hazard ratio of infected snails compared to non-infected snails was 1.65 (95% confidence interval: 1.35–1.99; *p* = 0.01).

**Figure 4:**
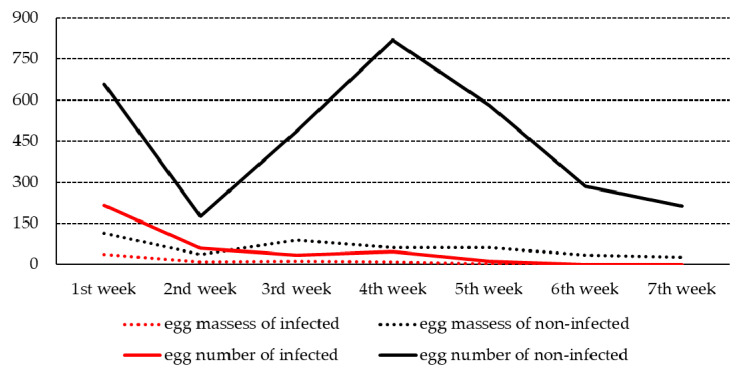
Fecundity of *Biomphalaria pfeifferi* snails that were infected or non-infected with *Schistosoma mansoni* (*p*-value < 0.001 for number of egg masses; *p*-value < 0.001 for number of eggs; solid line, number of eggs; dotted line, number of egg masses).

Figure 4 shows the fecundity of the infected and non-infected snails. The egg masses of the infected snails decreased gradually and reached their lowest number at the fifth week after infection. The total number of egg masses produced by infected snails was significantly lower than that produced by non-infected snails (70 vs. 473 egg masses). Similarly, the total number of eggs in the egg masses of infected snails was significantly smaller than that in the egg masses of the non-infected snails (368 vs. 3219 eggs).

**Figure 5:**
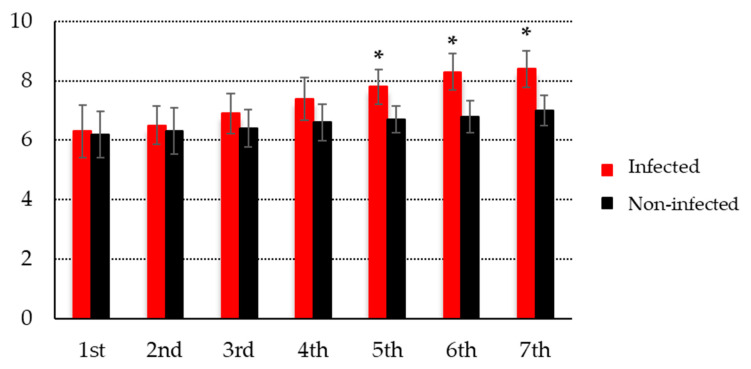
The size of *Biomphalaria pfeifferi* snails exposed to *Schistosoma mansoni* compared to the non-infected control group (*x*-axis, week; *y*-axis, mm; * *p*-value < 0.001).

Figure 5 illustrates the size of *B. pfeifferi* snails that were infected and non-infected with *S. mansoni*. The infected *B. pfeifferi* snails were significantly larger than the non-infected snails (8.4 (±0.6) vs. 7.0 (±0.5) mm at 7th week, *p* < 0.001).

## 4. Discussion

Although many existing studies in the literature have suggested the rhythmicity of schistosome cercaria, the exact peak time varies depending on the environmental conditions surrounding water bodies and host behaviors [18,19,20,21]. Information regarding the shedding pattern of schistosome cercariae in the White Nile River has not been well documented.

We detected a diurnal pattern of cercaria release, which peaked at 9:00–11:00 a.m. in the target areas of the White Nile River. The cercarial peak time of *S. haematobium* and *S. mansoni* is very similar to that found in the Blue Nile River in the Gezira and Blue Nile States and also in the Nile River around the Nuba Mountains [26,27]. The cercarial peak time of amphistomes and echinostomes was a bit earlier than that of schistosomes, which is consistent with previous findings [26].

The emission pattern of cercariae takes place at a time interval that optimizes transmission to the vertebrate host [28]. The rhythmic emergence of cercariae is synchronized by exogenous factors to which the cercariae respond. It is believed that these cercarial emergence rhythms are adaptive and that they have been selected because they enhance parasite transmission in the host [29]. Cercarial emergence behavior may differ between local populations of a trematode species when different hosts with different activity periods are involved in the transmission dynamics [30,31]. Overall, the shedding pattern of *S. haematobium* and *S. mansoni* occurs during the day for human infections [29,30,31]. However, intra-species diversity in the cercarial patterns of schistosomes was observed in West Africa [32]. First, the cercarial emergence pattern varied depending on the climatic and vegetal features of transmission areas. For instance, the time for cercarial shedding was significantly earlier in the shaded sites of the forest zone than in open sites of the savanna zone in West Africa, which indicates that variation in the cercarial emergence pattern results from parasites’ adaptation to different climatic and vegetal zones. In Puerto Rico, the highest density of cercariae in water was at noon in streams and at 4:00 p.m. in ponds [33]. In the Nile River and related canals in Egypt, the highest density of *S. haematobium* cercariae in water was observed between 7:00 and 9:00 a.m. [34]. A study done in an endemic area of *S. haematobium* in Kenya showed diurnal fluctuations of cercariae in ponds and rivers, with the highest density around noon [35]. Second, the pattern could also vary depending the host’s behavior, such as water contact time [36]. The behavior of the definitive hosts is associated with emergence rhythms [31]. The cercarial rhythm of emissions in Libreville, which peaked at 1:00 p.m., was explained by human behaviors in terms of contact with water, as people in this area most commonly came into contact with water to bathe between 11:00 a.m. and 4:00 p.m. [37]. In other areas, such as Benin, where water contact occurred most frequently for purposes such as artisan fishing, the cercarial emergence pattern had an ultradian pattern, with a primary peak found at midday and secondary peaks at dawn and dusk [38]. Cercarial shedding patterns also show seasonal variability. For instance, according to a study in a rural area in the Northern Province, South Africa, a peak in high-intensity shedding from *B. pfeifferi* was observed in the 9:00–11:00-a.m. period during the rainy season but in the 7:00–9:00-a.m. period in the dry season [39]. All in all, even though most of the existing literature points to a diurnal pattern of cercarial shedding of *S. haematobium* and *S. mansoni*, the shedding pattern may vary across areas within daylight hours depending on the circumstances of the surrounding water bodies and human behaviors. We thus assessed the distribution of cercarial shedding throughout the day in Sudan in order to develop a more tailored health strategy. People are recommended to avoid making contact with water at high-risk times for infection. Cercarial periodicity data also have practical applications for persons concerned with health education with respect to schistosomiasis. These data may also be of importance in designing a sustainable schistosomiasis-control program [40].

Infection significantly reduced the survival rates of *B. pfeifferi* compared to non-infected snails during 7 weeks after infection. The most snail deaths were observed from 4 to 6 weeks after infection. This peak in mortality might have been associated with a peak in cercarial shedding although we did not examine this possibility. Mechanical tissue damage, cercarial penetration, consumption of digested food, and toxic excretions from the parasite might be reasons for snail deaths [41]. The number of egg masses of infected snails decreased gradually and reached its lowest score in the fifth week after infection. The number of egg masses produced per infected snail was significantly smaller than that produced by non-infected snails. Similarly, the number of eggs per egg mass of infected snails was also significantly smaller than that of their counterparts.

The absence of any program for vector control is a key factor contributing to the spread of schistosomiasis infections [41,42,43]. Sudan is not an exception, and the lack of financial resources and trained personnel is the main reason for this absence.

The main natural asset of Sudan is the Nile River, which extends from the equatorial regions in the south to the deep desert in the north. It runs through many states of the country. In Sudan, irrigation schemes and agricultural development projects were properly designed for maximum production without consideration of the side effects in terms of environmental changes. Thus, the price has been paid for agricultural and economic development in the form of a considerable increase in both the prevalence and intensity of schistosomiasis [44,45,46,47]. Human contact with cercariae-infested water is a prerequisite for the transmission of schistosomiasis. There are a number of reasons for water contact in the target areas of this study. Some of the reasons are as follows based on our observations and key informants’ insights during the 1-year longitudinal survey: First, power is frequently off; specifically, electricity failures and corresponding maintenance-related problems of the water supply systems cause frequent interruptions of water from pipes. For this reason, the nearby residents have no alternative other than using infested water bodies for domestic and recreational purposes. Second, there are some specific cultural practices in the target areas: during funeral or birth ceremonies, large family groups visit the shore of the White Nile River and clean their bodies. In particular, during the rainy season, they tend to cover their bodies with silty soil from the shore and then wash with water in the river. Open defecation is also a key risk factor for schistosomiasis transmission in this area. The intermediate-host snails at these sites are, therefore, heavily exposed to large numbers of miracidia. All these behaviors lead to high levels of schistosomiasis transmission. Another concern about the high transmission of schistosomiasis in Sudan is the substantial population movement across States and borders. It was highlighted that intensive population movement has contributed to the high prevalence of schistosomiasis [5,6]. This type of large population movement tends to increase schistosomiasis transmission and makes it hard to adopt comprehensive control and elimination strategies. Although we did not directly survey individuals about water contact behaviors in this study, we believe that the cercarial shedding pattern might have evolved in accordance with host behaviors to make their transmission most successful. During daylight hours, particularly around midday, is time when farmers and fishers actively engage in their occupational work. Based on individual communications with key informants in the White Nile, we found an interesting result that women in the community had a lower prevalence of schistosomiasis, and they reported contacting water for defecation and urination after sunset, whereas men did so at various times of day. More research based on direct and careful observations of water contact behavior is warranted to identify the times of day when people most actively come into contact with water, which will help to formulate an adequate strategy for health education. The following are policy recommendations based on this study: First, the results should be reflected in the development of health education strategies. Particularly, if there is no other alternative in terms of water and sanitation improvement, community residents should be guided not to contact water during the intensive cercarial shedding time. Second, stakeholders in the public and private sectors, such as organizations in the agricultural and fishery sector and sugar cane companies, should be clearly informed of this, and they should strive to improve water and sanitation facilities for farmers, fishers, and sugar cane workers, who mainly work around infested water bodies, such as irrigation canals and rivers [48]. Third, it is necessary to develop an enabling environment to prevent people from coming into contact with infested water and to control snails in a sustainable manner. For instance, community residents can be mobilized to dig channels for water flow to interfere with snails’ survival by making water flow fast and removing plants. Creating laundry stations near water bodies with community participatory methods could also be a good way to prevent direct contact with infested water.

## 5. Conclusions

Since the FMOH of Sudan aims to transition from control to elimination of schistosomiasis, a range of interventions has been suggested, including the provision of safe water and improvements in sanitation, snail control, health education, and chemotherapy. Experience has shown that no single control method is likely to break the transmission cycle, and so all available methods should be considered and used concurrently. Thus, sound epidemiological surveys should be conducted to obtain empirical values of infection-related parameters of schistosomiasis. The global health community has recapitulated that there is a serious gap in information regarding biomedical and epidemiological aspects of schistosomiasis, including its vectors. An understanding of the dynamics of transmission, including the emergence pattern of cercaria and the reproduction, survival rate, and growth of infected snails, will be helpful for formulating schistosomiasis-control and -elimination strategies. Health education campaigns should incorporate information on cercarial rhythmicity by encouraging people to avoid water contact behavior around the peak time of cercarial emission. The fecundity, survival rate, and growth of infected snails are related to the competence of snails to serve as hosts for schistosomes. These can be used in schistosomiasis disease modeling as important parameters related to snail population dynamics. Understanding these dynamics of infected snails could help predict the snail population and, eventually, infection and re-infection of schistosomiasis.

## Data Availability

Data will be shared upon the request (hassanhassoon@hotmail.com).
